# Next-generation heparin antidotes: “Exosomes as a biocompatible alternative to protamine sulfate”

**DOI:** 10.1007/s11033-026-12145-7

**Published:** 2026-06-19

**Authors:** Leyla Bahar, Mehmet Erin Tüysüz, Pınar Hüner Omay, Vehbi Kınay, Kansu Büyükafşar, Sema Erden Ertürk

**Affiliations:** 1 Health Sciences Institute, Department of Stem Cell and Regenerative Medicine, Mersin University, Çiftlikköy Campüs, Mersin, Türkiye; 2 Department of Cardiovascular Surgery, Health Sciences University, Mersin City Training and Research Hospital, Mersin, Türkiye; 3 Onkim Stem Cell Technologies, İstanbul, Türkiye; 4 Faculty of Medicine, Internal Medical Sciences, Department of Pharmacology, Mersin University, Mersin, Türkiye; 5 Vocational School of Health Services, Mersin University, Mersin, Türkiye

**Keywords:** Heparin, Protamine, Exosome, Haemostasis, Clotting

## Abstract

**Purpose:**

Controlling bleeding is one of the most fundamental challenges in cardiac surgery. Bleeding is controlled by neutralising heparin with protamine. Our study investigates exosomes as an alternative treatment option to protamine for use in haemostasis.

**Methods:**

Rats were divided into 5 groups, with 4 rats in each group: Group 1 underwent puncture, Group 2 underwent heparinised puncture, Group 3 received exosomes prior to heparinised puncture, Group 4 received exosomes prior to heparinised puncture with heparin neutralised by protamine, and Group 5 received heparinised puncture with exosomes administered during puncture. Biochemical analysis was made, histopathological analysis showing tissue damage and Mast Cell Activity (MAST )in lung tissues. Western blot method for SERPINC1 expression.

**Results:**

Despite high Activated Clotting Time levels, bleeding time was found to be shorter in the group treated with exosomes. In MAST analyses, Group 4 had the highest density, while Group 3 had a density close to that of the control group. A significant difference was detected between Groups 3 and 4. in terms of lung tissue damage (*P* < 0.001). Tissue damage was most pronounced in Group 4. The application of exosomes significantly reduced inflammation and oedema in Group 3.

**Conclusion:**

Exosomes shorten bleeding time and reduce lung tissue damage when administered before puncture. Exosomes have the potential to replace protamine in the future.

**Supplementary Information:**

The online version contains supplementary material available at 10.1007/s11033-026-12145-7.

## Introduction

Systemic heparinisation, hypothermia, haemodilution, and effects on the coagulation and fibrinolysis systems during cardiac surgery performed with cardiopulmonary bypass (CPB) can lead to excessive bleeding and increase the need for blood transfusion [1]. Despite their adverse effects on the cardiovascular and haematological systems, heparin and protamine are indispensable agents in cardiac surgery. Heparin is used as an anticoagulant in open heart surgery. Protamine is administered to neutralise the effects of heparin after CPB surgery and causes hypotension in patients. The mechanism of this side effect is not fully understood [2]. When protamine binds to heparin, it can cause acute, reversible pulmonary hypertension [3].

Exosomes are nano-sized vesicles containing biological signalling molecules that mediate cell-to-cell communication. Compared to cells or synthetic nanoparticles, exosomes derived from cells have therapeutic potential as therapeutic agents, with remarkable biocompatibility/stability properties. Moreover, they have many advantages, including superior potential for loading with different cargoes [4, 5]. Today, many researchers are investigating the role of exosomes and biomolecules containing exosomes in the prevention, diagnosis, and treatment of clinical diseases, including cardiovascular diseases [6]. Exosomes play an important role in the pathogenesis of many diseases. Studies have shown that these vesicles, which can be obtained from all body fluids, regulate intercellular communication, signal transmission, genetic material transfer, and immune response. However, to the best of our knowledge, no studies have yet investigated the haemostatic effects of exosomes. Therefore, our study is novel and represents the first contribution in this field, with the potential to support the development of exosome-based approaches as an alternative to protamine in haemostatic applications.

## Methods

### Ethical statement

Ethical approval for our study (No. 12/07/03-2025) was obtained from the Saki Yenilli Laboratory for Experimental Animal Production and Application. The experimental animals were obtained from the same laboratory. Our study was conducted in accordance with the ARRIVE guidelines for reporting animal research. All experimental procedures involving animals were performed in compliance with relevant institutional, national, and international guidelines and regulations. Furthermore, informed consent was obtained from the mother of the newborn whose umbilical cord blood was used to obtain exosomes.

### Grouping of experimental animals

Animals and experimental design: The study included 5 male Wistar albino rats weighing 250–300 g in each group.

The rats were divided into 5 groups: All procedures were performed under anaesthesia. The groups were: Group 1 (Control Group): punctured, Group 2 (Heparin-Treated Group Only): punctured after heparinisation, Group 3 (Heparinised Group Given Exosomes Before the Procedure): punctured after heparinisation and given exosomes before the procedure, Group 4 (Heparinised Group with Exosomes Administered Before the Procedure): heparinised and given exosomes before the puncture, neutralised with protamine, Group 5 (Heparinised Group with Exosomes Administered During the Procedure): heparinised and given exosomes during the puncture.

### Exosome isolation method

The exosomes used in our study were obtained at the Istanbul Onkim Stem Cell Technologies centre. The umbilical cord blood of a healthy newborn baby was centrifuged to collect plasma. The collected plasma was centrifuged at 2000 xg for 10 min to remove erythrocytes. After centrifugation, 50 ml of plasma was separated into a falcon tube. The obtained plasma was passed through a 0.22 μm Polyethersulfone (PES) filter. The plasma was then passed through a Tangential Flow Filter. The filtration process took approximately 1 h. As a result of filtration, exosome particles ranging in size from 30 to 180 nm were obtained. Sterility, mycoplasma, immunophenotyping (CD81 and CD9 surface antigens), and particle count/size analyses were performed on the exosome samples. Concentrated exosome samples that met the acceptance criteria were flasked according to the desired particle count. Based on the particle count results of the concentrated exosome, 1 µl of sample contained 1 billion particles. After the sterility results were obtained, 5 µl of concentrated exosome was diluted with 5 ml of PBS and prepared in a flask. For the exosome application, 2.5 ml of exosome suspension was administered to each rat via the tail vein. The suspension used was prepared to contain 1 billion exosome particles per millilitre. A total of 2.5 billion particles/rat were administered in each application.

### Heparinisation process and measurement of Activated Clotting Time (ACT) levels

The heparin dose in rats was administered according to the study by Tuthill and colleagues [7]. The rats weighed an average of 250–300 g. For anticoagulation, 2 IU/g of heparin was administered. The administered heparin was neutralised with protamine sulphate at a ratio of 1:1.1; each rat was given 3 mg of protamine intravenously. Ten minutes before femoral artery puncture, except for the control group, the other rats were given an intravenous dose of Heparin (Nevparin^®^; Mustafa Nevzat) at 2000 IU/kg (on average 0.08 ml/rat heparin, diluted with 0.92 ml physiological saline). Blood samples were taken from all rats after heparin administration to measure the ACT. Measurements were performed using 2–3 ml of whole blood taken from the inferior vena cava of the rats. The blood was activated in a special tube or device. The blood clotting time was measured and recorded in seconds. The Medtronic ACT (Medtronic Inc. Parkway, Minneapolis USA) device was used for this purpose.

### Creation of a bleeding model and exosome application

Anaesthesia was achieved by intramuscular administration of 35 mg/kg ketamine + 5 mg/kg xylazine. The right groin area of the rat was shaved, and the skin and subcutaneous tissue were dissected. The Arteria and Vena Femoralis sheaths were made visible. The sheath was dissected, and the artery and vein were separated from each other. A 22-gauge cannula needle was inserted into the isolated femoral artery and perforated it. To control the bleeding and remove the blood from the area, another person applied pressure to the wound with a sponge for 10 s. The sponge was removed, and a standard pressure was applied by placing a 200 g fixed weight on the wound. From this point onwards, a stopwatch was started to determine the bleeding time. Pressure was applied to the artery for 600 s. Bleeding control was checked at two-minute intervals. Bleeding lasting longer than 600 s was recorded as uncontrolled bleeding. Group 1 underwent puncture only, while Group 2 underwent heparinised puncture. Group 3 received 2.5 ml of exosomes 60 min prior to the heparinised puncture procedure. Group 4 received 2.5 ml 60 min prior to the puncture procedure, with heparin neutralised with protamine. Group 5 received 2.5 ml of exosomes during the puncture procedure, with heparinisation. After the bleeding control period was completed, the rats’ abdomens were opened, the inferior vena cavae were explored, and the necessary blood samples were collected for the study. At this stage, onitor the effect of anticoagulant (blood-thinning) drugs such as heparin. Femoral Artery Exploration was performed on rats. The rats were then sacrificed and their femoral arteries were excised for Western Blot analysis. The rats’ lung tissues were removed for histopathological tissue examination. At the end of the study, all rats were sacrificed under anaesthesia and disposed of in special waste containers (Supplemental Data).

### Histopathological analysis

Rat lung tissues were fixed in a 10% formaldehyde solution, and routine tissue examination procedures were performed. Toluidine blue, which stains mast cells, was used to evaluate the reaction to heparin in the lung tissue [8]. Hematoxylin-Eosin (H-E) staining was applied to determine the tissue damage score [9].

### Biochemical analysis

Serums were mixed with the reagent and incubated (4 °C/30 min.), then centrifuged at 15,000×g for 2 min. The tests measured haemogram parameters and D-dimer (D = D) levels (https://www.ottosci.com/).

### Western blot analysis

The femoral arteries of rats were evaluated using the Western Blot method [10]. Tissue samples were homogenized in RIPA buffer containing protease inhibitors and centrifuged at 10,000 rpm for 10 min at 4 °C. Protein concentrations were determined using the Qubit 4 system. Samples (30 µg/µL) were mixed with Laemmli buffer, denatured at 70 °C for 10 min, separated by SDS-PAGE, and transferred onto a membrane at 110 V for 60–90 min.

Membranes were blocked with 5% BSA and incubated overnight at 4 °C with primary antibodies against β-actin and Serpin C1. After washing, membranes were incubated with secondary antibody for 1 h at room temperature. Protein bands were visualized using ECL substrate and imaged with the iBright 750 system.

### Statistical analysis

ACT levels between groups were compared using an independent t-test. Five independent groups were compared in terms of SERPINC1 Density. The distribution of continuous variables was assessed using the Kolmogorov–Smirnov test; as the assumptions were not supported, the non-parametric one-way Kruskal–Wallis H test was used for group comparisons. When significance was detected in the test, the Dunn post-hoc test was applied for multiple comparisons, and p-values were reported. The significance threshold was set at *p* < 0.05. For intergroup comparisons, analyses of the MAST (Mast Cell Activity) parameter were evaluated using the Kruskal–Wallis test. All analyses were performed using IBM SPSS software.

## Results

### Exosome isolation and characterization

**Fig. 1:**
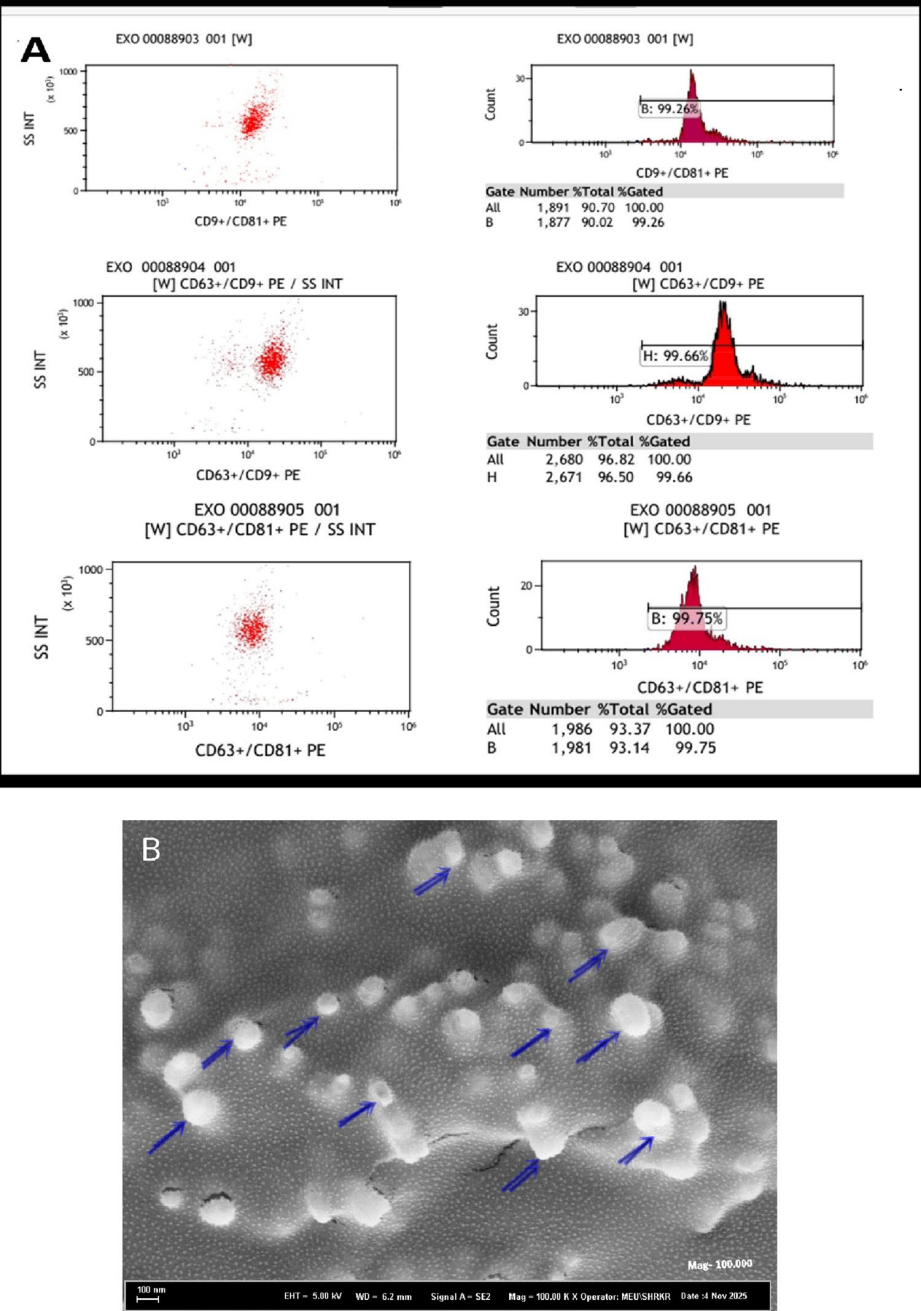
**A** Flow cytometry analysis demonstrated strong positivity for canonical exosome markers CD9, CD63, and CD81. Double-positive populations (CD9/CD81, CD63/CD9, CD63/CD81)exceeded 99%, supporting successful exosome isolation. **B** SEM analysis confirmed the presence of isolated exosomes with typical round-shaped vesicular morphology and nanoscale size distribution (scale bar: 100 nm)

Phenotypic characterization of the isolated extracellular vesicles by flow cytometry demonstrated high positivity for established exosomal markers CD9, CD63, and CD81. The proportions of double-positive vesicles were 99.26% for CD9/CD81, 99.66% for CD63/CD9, and 99.75% for CD63/CD81, indicating a highly enriched exosome population (Fig. 1A). Furthermore, SEM analysis demonstrated nanoscale vesicles with typical spherical morphology, further supporting the successful isolation of exosomes. These complementary analyses confirmed both the structural and immunophenotypic characteristics of the isolated exosomal vesicles (Fig. 1B).

### Western blot analysis

**Fig. 2:**
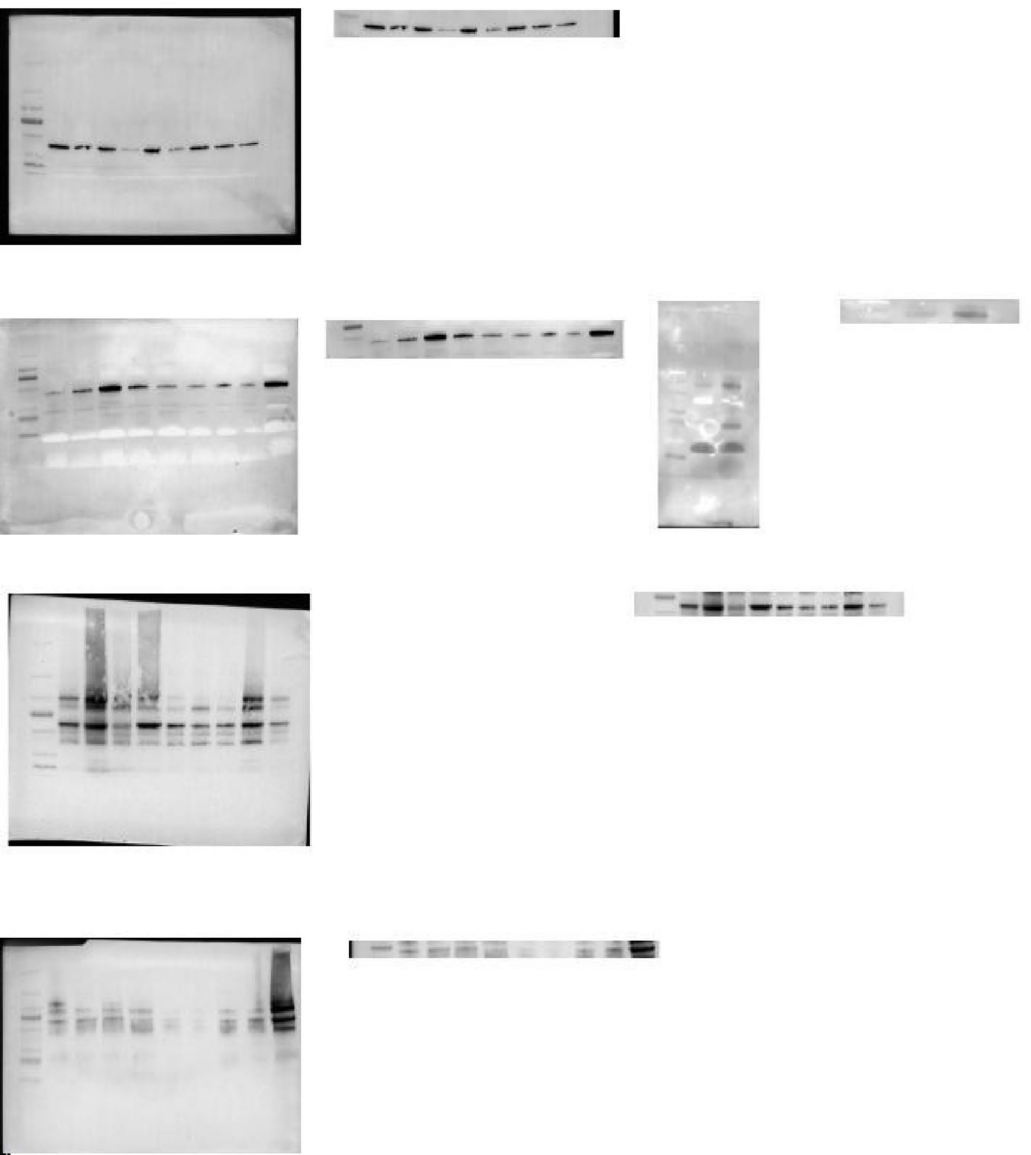
Examples of Western blot analysis of SerpinC-1 protein expression in experimental groups. Changes in SerpinC-1 band intensities are observed in the experimental groups. An equal amount of total protein was loaded into each well, and β-actin was used as a loading control. Band intensities were quantitatively analysed and normalised to the control group (*p* < 0.05 was considered statistically significant)

**Fig. 3:**
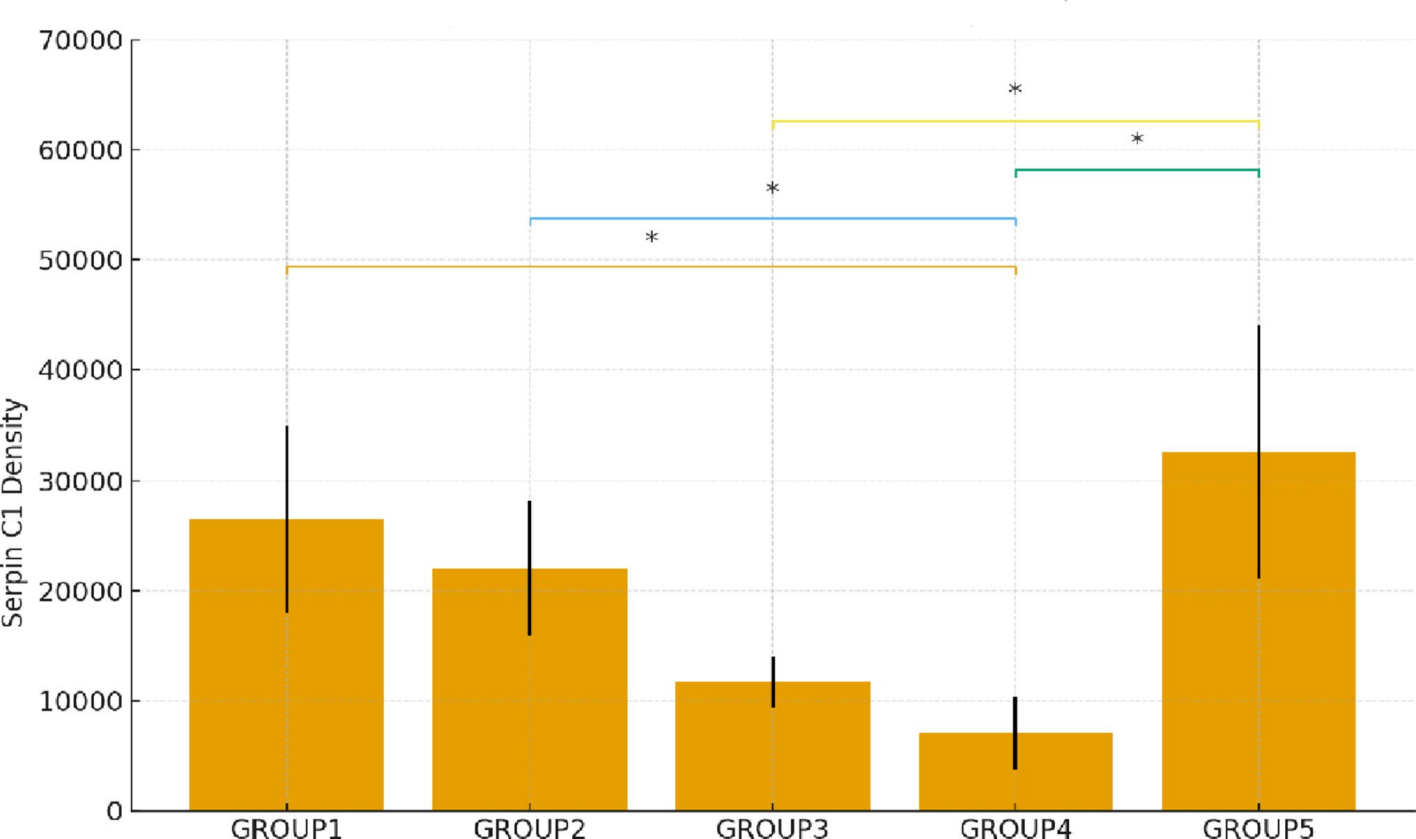
Comparison of SerpinC1 levels between groups. The lowest SerpinC1 level was detected in group 4

Normalized expression levels of serpin C1 differed between groups. Differences in mean values suggest potential group-dependent modulation of coagulation pathways. One-way ANOVA analysis of normalized serpin C1 expression levels yielded F = nan and p = nan values. SERPINC1 expression increased in the heparin-treated group, while this increase was balanced in the group receiving exosomes before treatment. In the protamine-treated group, SERPINC1 levels were significantly elevated, which was associated with increased tissue damage (Figs. 2 and 3). Other pairwise comparisons did not show significance after correction (*p* > 0.05).

### Evaluation of histopathological damage score

**Fig. 4:**
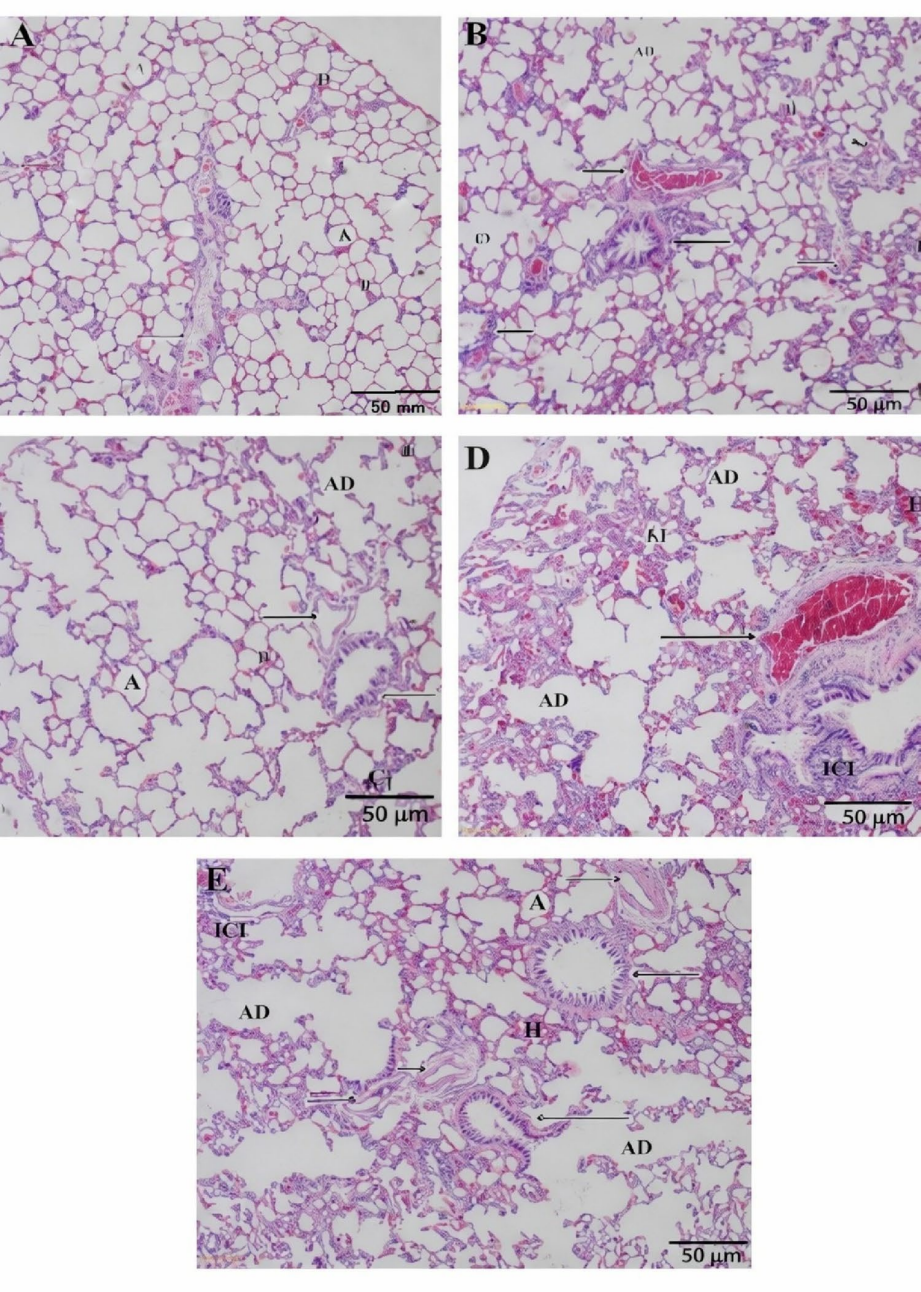
Hematoxylin–Eosin (H&E)-stained lung tissue sections from experimental groups (**A–E**). Group 1 showed minimal hemorrhage and mild inflammation with preserved alveolar septa. Group 2 exhibited prominent hemorrhage, moderate inflammation, mild-to-moderate edema, and slight septal thickening. Group 3 demonstrated reduced hemorrhage, inflammation, and edema compared with Group 2, with largely preserved architecture. Group 4 showed severe inflammation, edema, and marked septal thickening, indicating extensive tissue damage. Group 5 displayed moderate hemorrhage, inflammation, and septal thickening, suggesting a weaker protective effect than Group 3. A: alveolus; AD: alveolar duct; ICI: inflammatory cell infiltration; H: hemorrhage. Arrows indicate arterioles (right) and bronchioles (left). H&E stain, ×20; scale bars = 50 μm

**Fig. 5:**
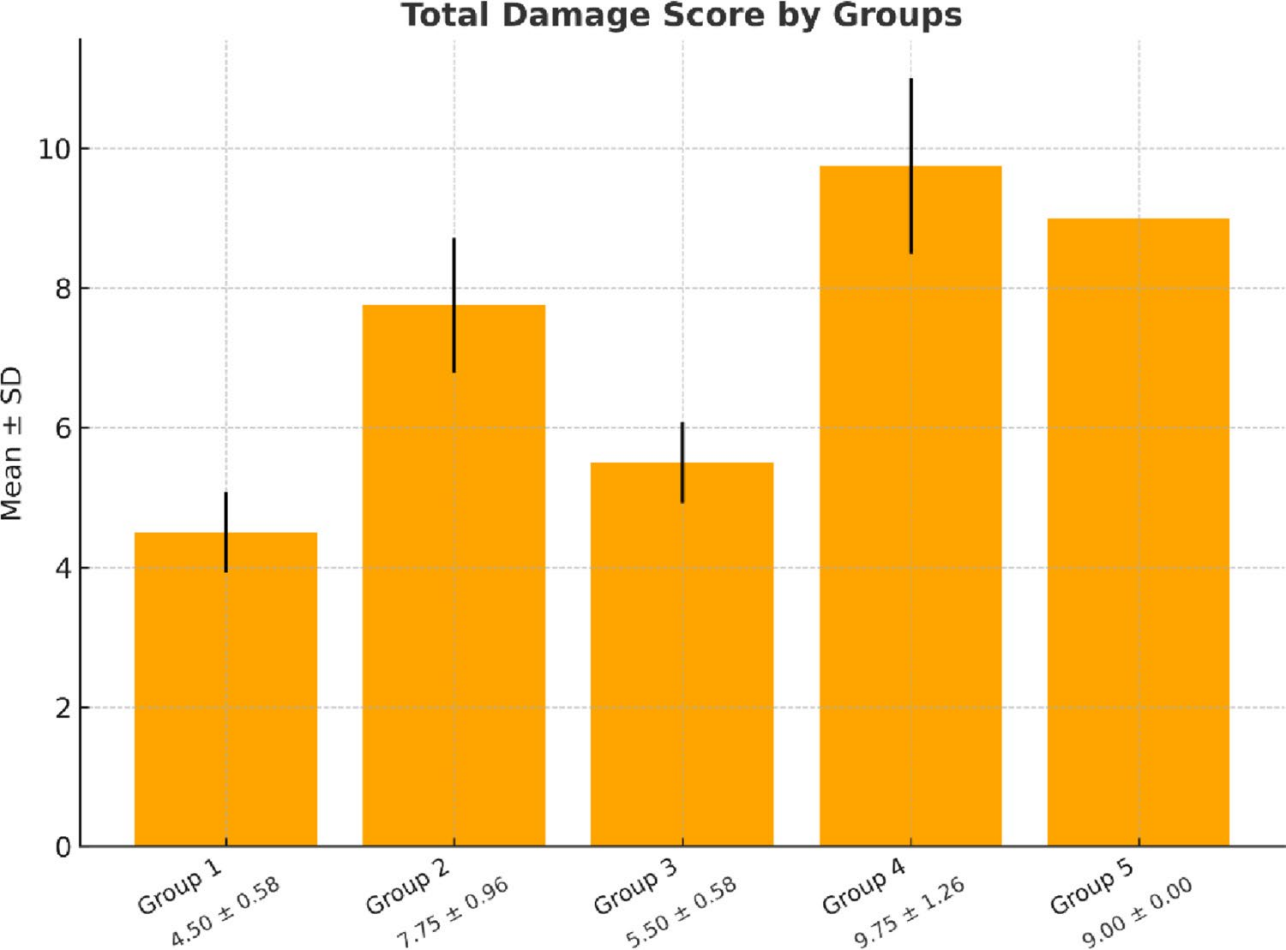
Total damage score in lung tissue by groups (Mean ± SD). Comparison of total lung injury scores among experimental groups. Histopathological damage was assessed by four parameters (inflammation, hemorrhage, edema, and alveolar septal thickening), each scored from 0 to 4. Groups 1 and 3 exhibited significantly lower injury scores compared with Groups 4 and 5. Data are presented as mean ± SD, with error bars indicating standard deviation

In light microscopic evaluation, lung tissue sections stained with H&E were examined for damage score (haemorrhage, infiltration, oedema, alveolar septal thickening). In Group 1, which was exposed to puncture trauma, minimal haemorrhage and oedema were observed histopathologically. Inflammatory cell infiltration was mild, and alveolar septal thickening was not prominent. Overall, the alveolar architecture was preserved, and tissue integrity was best preserved compared to the other groups. In Group 2, prominent haemorrhagic areas were noted. Inflammatory cell infiltration was greater than in the control group, and oedema was moderate. Alveolar septal thickening also increased compared to the control group. In Group 3, haemorrhagic areas associated with heparin were observed; however, the exosome application was found to significantly reduce inflammation and oedema. Alveolar septal thickening was also limited. This group was considered the best preserved histologically after the control group. In Group 4, contrary to expectations, tissue damage was most pronounced. It was characterised by widespread alveolar septal thickening, marked oedema and inflammatory cell infiltration. Haemorrhage and infiltration were prominent, with alveolar integrity and tissue architecture being disrupted. Consequently, the most unfavourable histological damage was observed in Group 4. In Group 5, although the inflammatory response was partially reduced by the administration of exosomes during puncture, haemorrhagic areas and oedema remained prominent due to heparin administration. Alveolar septal thickening was moderate (Figs. 4 and 5; Table 1).

**Table 1:** Lung tissue damage score groups comparison

Comparisons	Groups (mean ± SD)		*p*-value
Group 1 vs. Group 2	4.5 ± 0.58	7.75 ± 0.96	*P* = 0.0003
Group 1 vs. Group 3	4.5 ± 0.58	5.5 ± 0.58	*P* = 0.4983
Group 1 vs. Group 4	4.5 ± 0.58	9.75 ± 1.26	*P* < 0.001
Group 1 vs. Group 5	4.5 ± 0.58	9.0 ± 0.0	*P* = 0.0000
Group 2 vs. Groups	7.75 ± 0.96	NS	*P* > 0.05
Group 3 vs. Group 4	5.5 ± 0.58	9.75 ± 1.26	*P* < 0.001
Group 3 vs. Group 5	5.5 ± 0.58	9.0 ± 0.0	*P* = 0.0001

Statistically significant groups comparison (*p* < 0.05) are shown with their mean ± SD values. Post-hoc pairwise comparisons (Bonferroni adjusted) NS: Not significant after Bonferroni correction

### Analyses of the MAST parameter

**Fig. 6:**
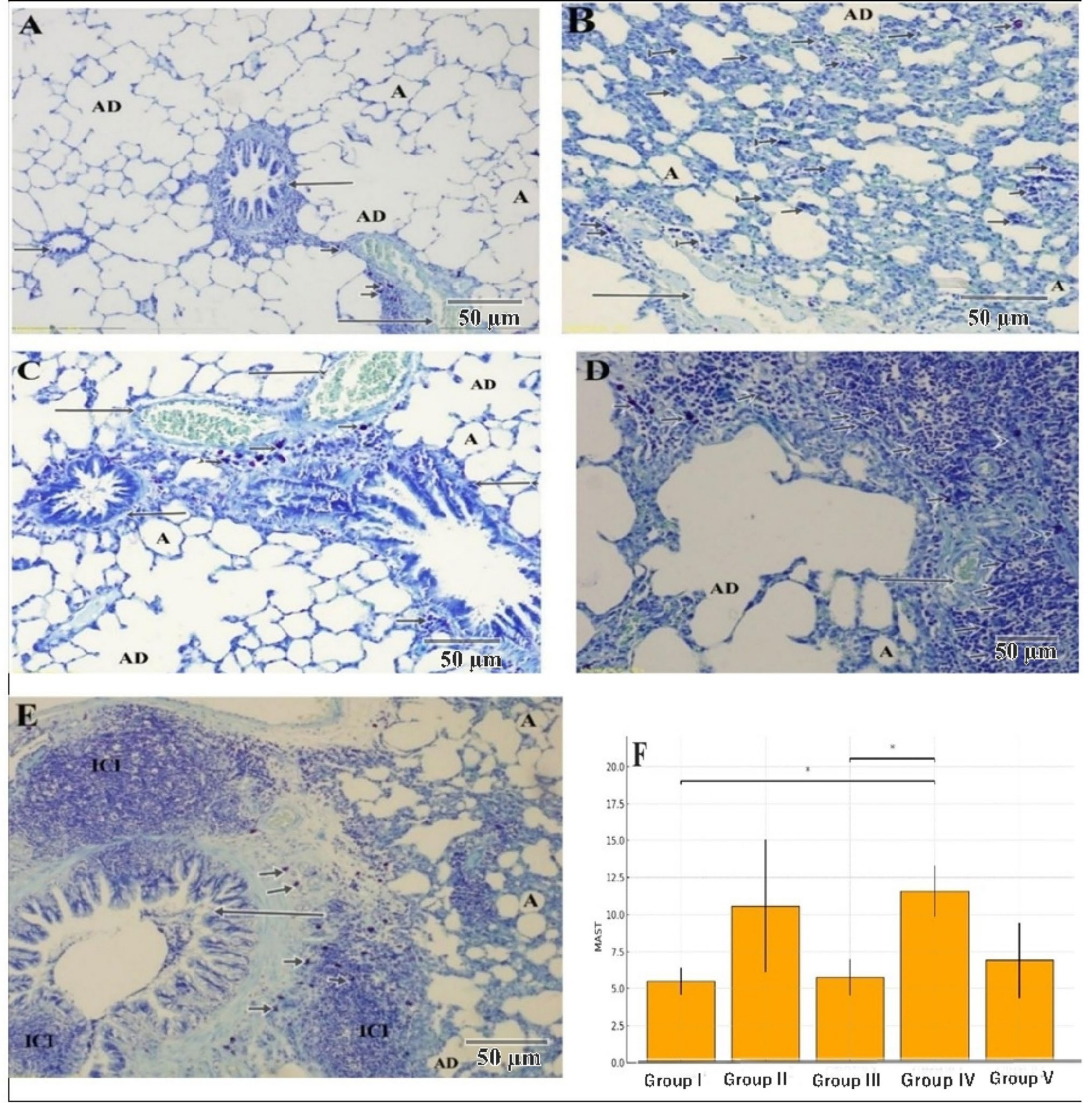
Sections of lung tissue showing MAST in different experimental groups (Fig. 6**A, B, C, D, E**). *“MAST levels were highest in Group 4 and significantly greater than in the control group and Group 3* (*p* < 0.05). Overall, exosome administration appeared to suppress mast cell infiltration, whereas this effect was lost after protamine neutralization. MAST in lung tissue. Mast cell density was highest in groups 2 and 4 (Fig. 6F). A: Alveolus, AD: Alveolar Ductus, right arrow: Arteriole, Left arrow: Bronchiole, Right short arrow: Mast cell. ICI: Inflammatory cell infiltration. Toluidine blue-stained, X20; Scale bar: 50 µm

In intergroup comparisons, analyses of the MAST parameter were statistically significant (*p* = 0.049). Following the Kruskal–Wallis test, two-tailed post hoc multiple comparisons revealed a statistically significant difference between Group 1 and Group 4 (*p* = 0.014). This difference indicates that MAST levels observed in Group 4 were significantly higher than those in Group 1. A significant difference was also detected between Group 3 and Group 4 (*p* = 0.018). This situation indicates that MAST in Group 4 has higher values compared to Group 3. These results suggest that the Group 4 application may have a pronounced stimulatory effect on MAST. No significant difference was observed in other group comparisons (*p* > 0.05) (Fig. 6).

The MAST in the lung tissue showed significant differences between the groups. In Group 1, a small number of mast cells were detected; the density was significantly lower than in the other groups. The number of mast cells increased significantly in Group 2, which was found to be significantly higher than in the control group (*p* < 0.05). Group 3 exhibited a low MAST, which remained at a low level and followed a similar course to the control group. In Group 4, MAST was observed at the highest level; this increase was statistically significant compared to both the control group and Group 3 (*p* < 0.05). In Group 5, MAST was higher than İn Groups 1 and 3, but no statistically significant difference was detected. Overall, it was observed that exosome application suppressed mast cell infiltration, but this effect disappeared after neutralisation with protamine (Fig. 6F).

### Evalution of statistical analysis

There is a moderate negative correlation between ACT level and platelet count; as ACT level increases, platelet count decreases (*r* = -0.466, *p* = 0.038). ACT levels between groups were compared using an independent t-test. The increase in ACT levels in the other groups compared to the control group was found to be significant (*p* < 0.05). The Kruskal–Wallis test was significant for SerpinC1 concentration (*p* = 0.004). In Dunn–Bonferroni post-hoc pairwise comparisons, comparisons between group 1 and group 4 (*p* = 0.005), group 4 and group 5 (*p* = 0.001), group 4 and group 2 (*p* = 0.017), and group 3 and group 5 were found to be significant (*p* = 0.017). Pearson’s and Spearman’s analyses showed no significant correlation between SERPINC1 expression and mast cell activity ((*r* = 0.09, *p* = 0.71).

## Discussion

In cardiovascular surgery, haemostasis mechanisms play a vital role. Our study introduces a new agent that will affect these mechanisms. Our study examines the effect of exosomes on haemostasis. Our findings are as follows: ACT levels were higher in the other groups compared to the control group. In MAST analyses, a significant difference was detected between Group 1 and Group 4, and between Group 3 and Group 4. The mast cell activity levels observed in Group 4 were higher than those in Group 1, and the MAST in Group 4 was higher than that in Group 3. As the ACT level increased, the platelet count decreased. Comparisons of SerpinC1 density between Group 1 and Group 4, Group 4 and Group 5, Group 2 and Group 4, and Group 3 and Group 5 were found to be significant. In terms of lung tissue damage, the exosome application significantly reduced inflammation and oedema in Group 3. This group was considered the best preserved histologically after the control group. Tissue damage was most pronounced in Group 4.

Systemic hemodilution and hypothermia applied during cardiac surgery with CPB significantly affect the heparinization, coagulation, and fibrinolysis systems, causing excessive bleeding and increasing the need for blood transfusion [1]. Since the development of CPB, unfractionated heparin has been the most commonly used anticoagulant for over 70 years [11].The reversible dose of protamine is estimated by considering the initial loading dose of heparin and the additional heparin doses administered regularly during CPB.

Several studies have reported that the reversible ratio of protamine to heparin ranges from 0.5 to 1.3. Because estimated heparin concentrations do not reflect real-time heparin levels, protamine dosing carries inherent risks, potentially leading to hemodynamic instability, coagulopathy-associated bleeding, inadequate reversal, or heparin rebound. Separate dosing of heparin and protamine after measuring heparin concentrations can mitigate complications arising from inappropriate dosing. In this context, Bull et al. developed the concept of heparin dose-response-based heparin dosing [12, 13]. ACT, introduced by Hattersley in 1966, has become a standard treatment modality for the follow-up of heparin anticoagulation [14]. Exosomes play a role in many physiological and pathological processes. Exosomes regulate biological functions by transporting lipids, proteins, and nucleic acids directly to target cells, and nucleic acids play a crucial role in this process [15]. Controlling bleeding is one of the most fundamental challenges in cardiac surgery. Bleeding is controlled by neutralising heparin with protamine. Complications arising during this neutralisation process occur both in cases of insufficient neutralisation and excessive protamine doses. Moreover, in patients exposed to protamine a second time, these reactions can be more severe and may also cause problems related to heparin resistance. Unfortunately, alternative treatments to protamine have not yet been developed. Furthermore, the absence of bleeding at high ACT levels is desirable in the early period following cardiovascular surgical procedures. A treatment capable of achieving this may be effective in preventing thrombosis of grafts and mechanical heart valves in the early postoperative period. Therefore, the development of new therapeutic approaches is essential to overcome these challenges. In our study, we sought to demonstrate that alternative exosome applications to protamine could achieve this goal. In our study, we found that bleeding stopped and bleeding time was shorter in rats treated with exosomes, while ACT levels remained high. These results suggest that exosome applications could be groundbreaking in preventing early graft thrombosis, which is desirable in cardiac surgery. Individualized heparin and protamine administration is a widely accepted strategy for reducing coagulation activation and bleeding complications. Although a link has been established with increased heparin requirements during CPB, the effect of protamine administration is unclear [16]. Protamine administration can lead to systemic reactions. Antigen-antibody responses to protamine sulfate trigger a type I anaphylactic reaction, which can range from hemodynamic instability such as tachycardia and transient hypotension to cardiovascular collapse, particularly during cardiac surgery. Given the high density of histamine-secreting mast cells or basophils in the airway and lung mucosal tissues, protamine’s ability to trigger histamine release has been investigated. However, in vitro studies have failed to demonstrate this effect at the standard dose commonly used in the clinic [17]. Protamine can also cause pulmonary vasoconstriction, bradycardia, and hypotension, leading to IgE-independent anaphylactoid reactions. Anaphylactic reactions to protamine can lead to life-threatening right ventricular dysfunction and pulmonary hypertension [18]. Exosomes can also engage in intercellular communication directly through their surface proteins. In another study, it was observed that exosomes interact with other cells through their surface proteins, potentially modulating antigen presentation and immune regulation processes in the immune system. Exosomes may also play a role in direct intercellular communication via surface proteins. Another study has identified the potential of exosomes to modulate antigen presentation and immune regulation processes in the immune system by interacting with other cells via surface proteins [19, 20]. In our study, analyses were also performed on MAST in the lung tissue of the experimental groups. A statistically significant difference was found between Group 1 and Group 4. This difference indicates that the levels of MAST observed in Group 4 were significantly higher than those in Group 1. This finding is an expected result in Group 4, where heparin-protamine was used. However, when comparing Group 3 with Group 4, the higher MAST levels in Group 4 compared to Group 3 indicate that the group administered exosomes has a higher MAST than the group treated with protamine. These results suggest that Group 4 treatment may have a significant stimulatory effect on MAST. As is known, exosomes are effective in potential immunoregulatory processes. Our study demonstrates that exosomes may reduce the likelihood of anaphylactic reactions associated with heparin-protamine use, in addition to their haemostatic effect. This is because exosome administration eliminates the need for protamine, which is used to neutralise heparin. Furthermore, the histopathological findings in Group 3 indicate that exosomes may play a protective role on lung tissue when used prior to the procedure. During our study, one rat in Group 4 experienced shallow breathing followed by cardiopulmonary arrest and death, while the other rats in the same group developed tachypnoea, consistent with anaphylaxis findings. Our histopathological findings also support this. These findings suggest that the combined use of exosomes and protamine to stop bleeding may be inadvisable.

Haemostasis is a complex process involving a series of coagulation factors. The intrinsic pathway consists of factors II, IX, X, XI and XII, named fibrinogen, prothrombin, Christmas factor, Stuart-Prower factor, plasma thromboplastin and Hageman factor, respectively. The extrinsic pathway consists of factors I, II, VII, and X. Factor VII is called the stabilising factor [21]. The common pathway consists of factors I, II, V, VIII, and X. The factors circulate in the bloodstream as zymogens and are converted into serine proteases. These act as catalysts to cleave the next zymogen into more serine proteases, ultimately activating fibrinogen. Serine proteases, which are coagulation factors, include factors II, VII, IX, X, XI, and XII, while factors V, VIII, and XIII are not classified as serine proteases. The intrinsic pathway is activated via exposed endothelial collagen, while the extrinsic pathway is activated via tissue factor released by endothelial cells following external injury [22, 23]. It is known that heparin exerts its effect via the intrinsic system. Platelet counts also decrease with heparin administration. Protamine, being the antidote to heparin, indirectly affects the intrinsic system. However, the effect of exosomes on the bleeding cascade is unknown. In our study, the high ACT value and the presence of clotting suggest that the possibility of exosomes acting through the intrinsic system is weak. The absence of a statistically significant difference in Mean Platelet Volume (MPV) and platelet count between groups in our study suggests that exosomes activate the extrinsic system by behaving like tissue thromboplastin in the bleeding cascade. If this information is confirmed, exosomes could be used as an antidote to warfarin, which affects the extrinsic system. As our study is a pilot study, we aim to measure the levels of these factors in exosome applications and demonstrate their relationship with the heart-lung machine in our subsequent studies.

Antithrombin III(AIII) is an important plasma glycoprotein belonging to the serpin superfamily. It regulates the proteolytic activity of procoagulant proteases in the coagulation system [24]. The primary targets of antithrombin are thrombin, FXa, and FIXa. It can also inhibit FXIa, FXIIa, and TF-FVIIa.24 Two important structural features involved in the regulatory function of antithrombin are a reactive central ring that binds to the active site of coagulation proteases, trapping them in inactive covalent complexes, and a fundamental D-helix that binds to therapeutic heparins and heparan sulphate proteoglycans in vascular endothelial cells. Binding of the D-helix of antithrombin by therapeutic heparins enhances serpin reactivity with coagulation proteases via serpin conformational activation and a template (bridging) mechanism [25, 26]. SERPINC1, also known as AIII, is a plasma protease inhibitor and a member of the serpin superfamily. This protein inhibits thrombin and other active serine proteases of the coagulation system and regulates the blood coagulation cascade. Over 120 mutations have been identified for this gene, and most of them are known to cause AIII deficiency [27, 28]. Hereditary AT deficiency is the most severe type of thrombophilia with marked clinical heterogeneity requiring indefinite anticoagulation [29].

Recent studies have also focused on the potential applications of mesenchymal stem cell-derived exosomes in the treatment of disseminated intravascular coagulation, in addition to hemorrhage [30]. Innovative pharmacological and non-pharmacological strategies in coagulation management are examined, and the limitations of protamine and alternative approaches are discussed [31]. Exosomes may exhibit procoagulant activity through phosphatidylserine exposure and tissue factor expression, promoting thrombin generation Additionally, exosomes interact with platelets and endothelial cells, influencing haemostatic balance. On the other hand, mesenchymal stem cell-derived exosomes have been shown to exert anti-inflammatory and immunomodulatory effects [32, 33]. (Mackman, 2011). Kalluri & LeBleu, 2020). Since extracorporeal circulation was not used in our study, the inflammatory effect of exosomes was not investigated. However, the effect of exosomes was to reduce inflammation in Group 3. It did not reduce the allergic effect of heparin-protamine in tissues in Group 4.

In our study, the presence of SERPINC1 in vascular tissue was detected in the control group (Group 1) using the Western Blot method. In Group 4, a decrease in expression was observed compared to other groups due to the utilisation of SERPINC1 by the heparin-protamine-antithrombin 3 complex in the coagulation cascade. However, the absence of a difference in SERPINC1 levels between Groups 3 and 5, which received exosome treatment, and Group 1 suggests that exosomes exert their effects on coagulation without influencing SERPINC1 levels. Nevertheless, the timing of exosome administration resulted in a significant difference in SERPINC1 expression between Groups 3 and 5. This difference may be attributable to mutational variations in the SERPINC1 gene. The lower SERPINC1 expression, MAST, and lung tissue damage score observed in Group 3 compared to Group 5 may provide insight into the optimal timing of exosome administration. We believe that administering exosomes prior to the puncture procedure would be more appropriate.

## Conclusion

Our findings suggest that exosomes modulate coagulation independently of SERPINC1 while simultaneously attenuating protamine-associated pulmonary inflammatory effects, indicating their potential as a promising alternative to conventional haemostatic strategies in cardiac surgery. To further validate this, better designed and sufficiently powered randomised trials are required.

## Supplementary Information

Below is the link to the electronic supplementary material.

Supplementary Material 1

## Data Availability

The data for this study can be obtained from the corresponding author upon appropriate request.
