# Supplementary figures and images for: Next-generation heparin antidotes: “Exosomes as a biocompatible alternative to protamine sulfate”
Source: Mol Biol Rep. 2026 Jun 19;53(1):966. doi: 10.1007/s11033-026-12145-7 (PMC13282254; doi:10.1007/s11033-026-12145-7)

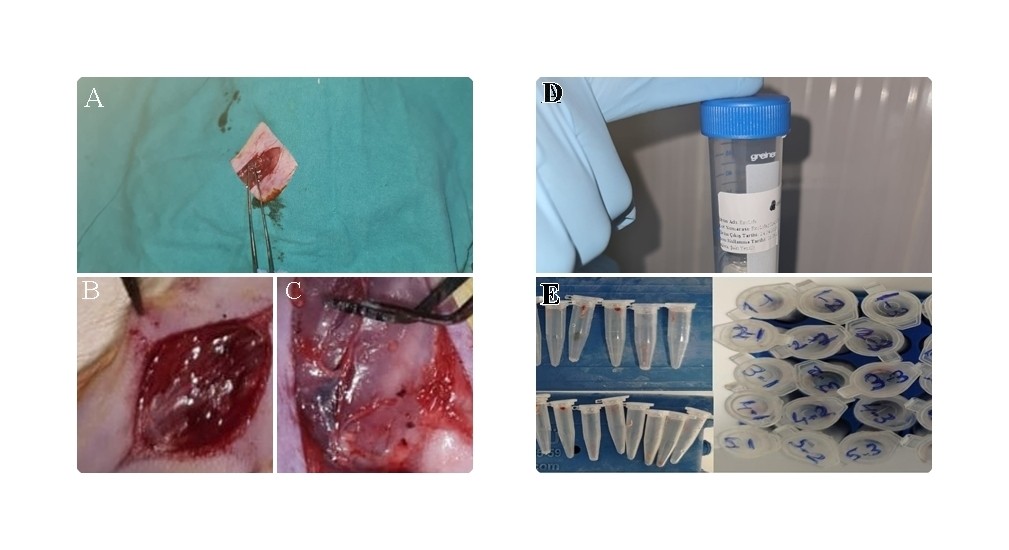

Supplement: Supplementary file 1 — Supplementary Material 1 [file 11033_2026_12145_MOESM1_ESM.jpg]
